# Sindbis virus neutralising antibodies detected in Swedish horses

**DOI:** 10.1016/j.onehlt.2021.100242

**Published:** 2021-03-25

**Authors:** Agnes Björnström, Anne-Lie Blomström, Manish Chandra Singh, Jenny C. Hesson

**Affiliations:** a Department of Medical Biochemistry and Microbiology, Zoonosis Science Center, Uppsala University, Sweden; b Department of Biomedical Sciences and Veterinary Public Health, Swedish University of Agricultural Sciences, Box 7028, 750 07 Uppsala, Sweden

**Keywords:** Alphavirus, Serology, Equine encephalitis, Equine fever, Arbovirus, Neutralisation test

## Abstract

A number of viruses transmitted by mosquitoes are well known to cause disease in both humans and horses, ranging from mild fevers to mortal neurological disease. A recently discovered connection between the alphavirus Sindbis virus (SINV) and neurological disease in horses in South Africa initiated this serological study in northern Europe, where the same genotype of SINV (SINV-I) is also highly endemic. We tested 171 serum samples, originally obtained from horses for other reasons from April to October 2019, for presence of SINV neutralising antibodies using a plaque reduction neutralisation test (PRNT). The serum from six horses reduced the plaque count more than 80%, and two out of these reduced the plaque count more than 90%. These horses were sampled in six different regions of Sweden, and included individuals sampled from April to August. This study shows that horses in Sweden have become infected with SINV and developed neutralising antibodies. Potential connections between infection and development of disease are important questions for future studies.

## Highlights

• SINV is a arbovirus that has been associated with disease in horses in South Africa.
• SINV is common in Sweden but any connection to equine infection is unknown.
• In this study, neutralising antibodies against SINV were detected in Swedish horses.
• This is the first study to indicate SINV infection in European horses.
• The clinical impact of SINV on horses will be an important focus for future studies.

## 1 Introduction

Vector-borne infections have a great impact on both human and animal health worldwide [1]. The involvement of vertebrate host/s and invertebrate vectors contribute to the complex transmission patterns of these infections, and new knowledge is constantly being added. Some arthropod-borne viruses (arboviruses), transmitted by mosquitoes, are well known to cause disease in both humans and horses. These include the alphaviruses; Venezuelan equine encephalitic virus (VEEV). Eastern equine encephalitis virus (EEEV), Western equine encephalitis virus (WEEV), all present in North America, Gheta virus (GV) present predominantly in Asia, and Ross River virus (RRV) present in Australia, as well as the flaviviruses; West Nile virus (WNV) and Japanese encephalitis virus (JEV). Many of these viruses target the central nervous system in both humans and horses, with neurological symptoms as a consequence, sometimes with mortal outcome [[2], [3], [4]] However, for RRV the symptoms in both horses and humans mainly come from inflammation of the joints, and GV is mainly associated with a febrile disease [[5], [6], [7]].

In northern Europe, the mosquito-borne alphavirus Sindbis virus (SINV) occasionally causes disease in humans, manifesting as fever and arthritis that could last for years [8,9]. SINV originates from Africa and has likely been introduced to northern Europe in the 1920s, potentially by migrating birds [10]. Recently, SINV has been linked to neurological disease in horses in South Africa [11]. In a study of 623 horses with unexplained febrile illness and/or neurological symptoms, eight horses tested positive for SINV RNA. Of the infected horses, one died from neurological disease, two survived neurological disease, and three recovered from a febrile illness. One of the horses also had mild colic and dysphagia from tongue paralysis. The remaining two horses also died, but was co-infected by WNV, thus the cause of death could not be established.

SINV has a wide distribution and has been detected in mosquitoes and birds across the Old World. It has been categorised into six genotypes, with genotype I (SINV-I) being associated to disease in humans [12]. SINV-I is distributed across Europe and Africa, but human cases are mainly reported from South Africa, Finland and Sweden [10]. Potentially, ecological factors such as vector abundance and host population dynamics, are important determinators of transmission intensity and influence the geographical distribution of disease cases [13].

Like many of the above mentioned arbovirues, SINV is transmitted by mosquitoes mainly from the genus *Culex*, and utilizes passerine birds as its natural host. In Sweden, it is well established that SINV-I is frequently being transmitted during the summer months, based on investigations of virus detection in the mosquito population and serological investigations of the passerine bird population [14,15]. If horses are susceptible to SINV-I and at risk of developing neurological disease, as been reported from South Africa, it is possible that northern European horses are also at risk of becoming infected with the virus. Since the link between SINV and infection in horses is new, the aims of this investigation were double: to initiate the study of SINV serology in horses, and to examine whether Swedish horses have been infected with SINV.

## 2 Materials and methods

**Fig. 1:**
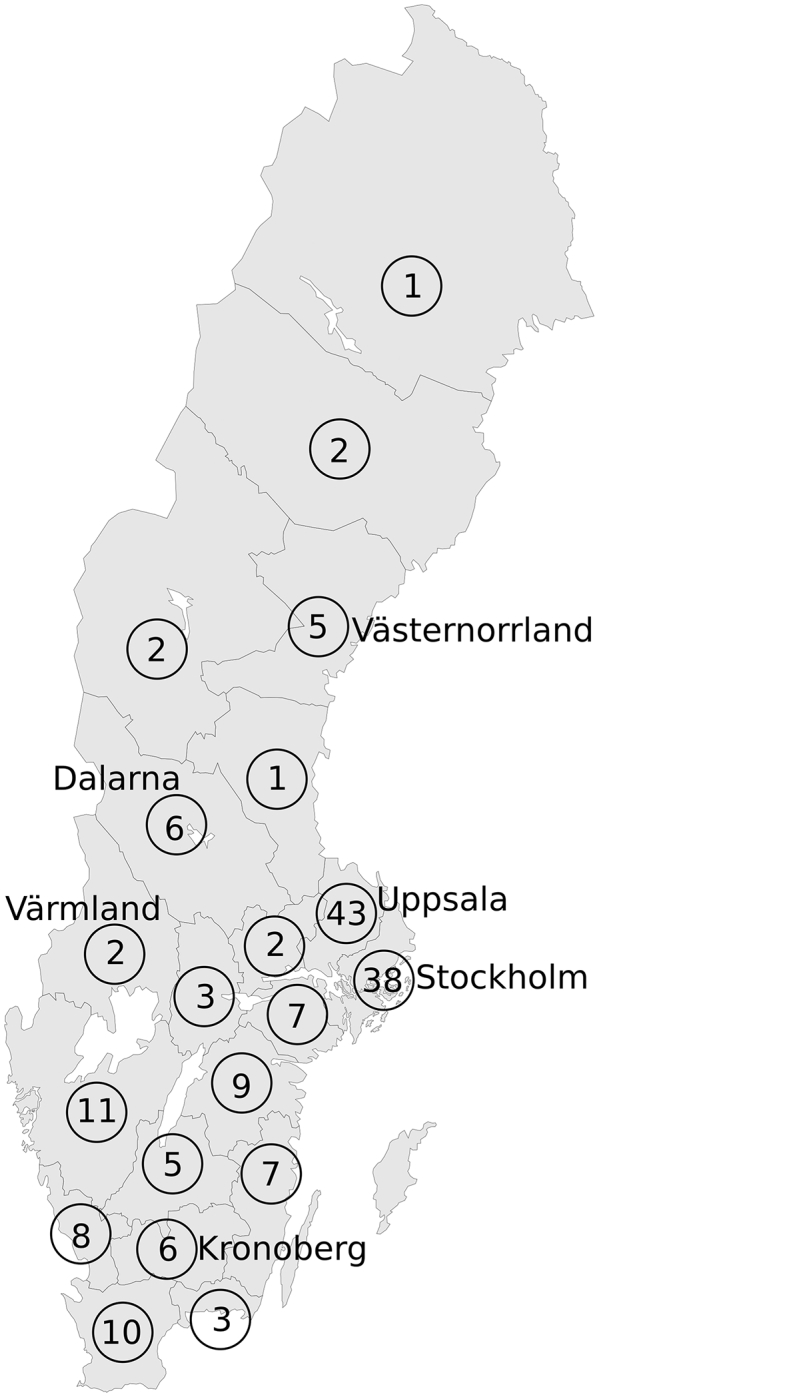
Sampling regions of horses analysed for neutralising antibodies against SINV. Circles show number of horses sampled in that region. Regions where horses had a titer of neutralising antibodies of 80% or more (≥PRNT_80_) are written out for comparison with Table 2.

In total, 171 serum samples from horses were used for testing the presence of neutralising antibodies against SINV. None of the samples were originally obtained for SINV diagnostics but taken for other purposes. All serum samples were obtained through the University Animal Hospital in Uppsala, Sweden, coded with only date and site of sampling. The serum samples included in this study were taken from horses between April 18th to October 10th, 2019, from 20 regions in Sweden (Fig. 1). The majority of samples came from regions Uppsala and Stockholm.

All samples were analysed by a SINV plaque reduction neutralisation test (PRNT) using a 20× serum dilution and SINV-I strain 09-M-358-5 , previously isolated from mosquitoes collected in central Sweden [[14], [15], [16], [17]]. Samples, negative control (serum from a lab rabbit), positive control (serum from a human SINV infected patient) and virus were all diluted in HBSS supplemented with 2% PenStrep and 2% HEPES (Thermo Fischer Scientific; Vilnius, Lithuania). All sera were heat-inactivated at 56 °C for 30 min prior to testing.

Each heat-inactivated serum sample was mixed with an equal amount of virus dilution and incubated at 37 °C for 1 h to allow any antibodies to react with the virus. After incubation, samples were inoculated in duplicates onto confluent Vero cells in a 24-well plate. Six wells per testing round were inoculated with the same end-dilution of the virus as was present in the samples, although in absence of serum. Cells were then incubated at 37 °C for 1 h to allow infection, before being overlaid with 1,5% Agar Noble (Sigma-Aldrich, MO, USA) which was mixed with equal amount of 2xMEM (supplemented with 8% FBS, 2% Penstrep and 2% Hepes) (Thermo Fischer Scientific; Vilnius, Lithuania), and 1% DEAD-dextran hydrochloride (Sigma-Aldrich, MO, USA). The agar was allowed to stiffen before the cells were returned to incubate at 37 °C over night.

**Fig. 2:**
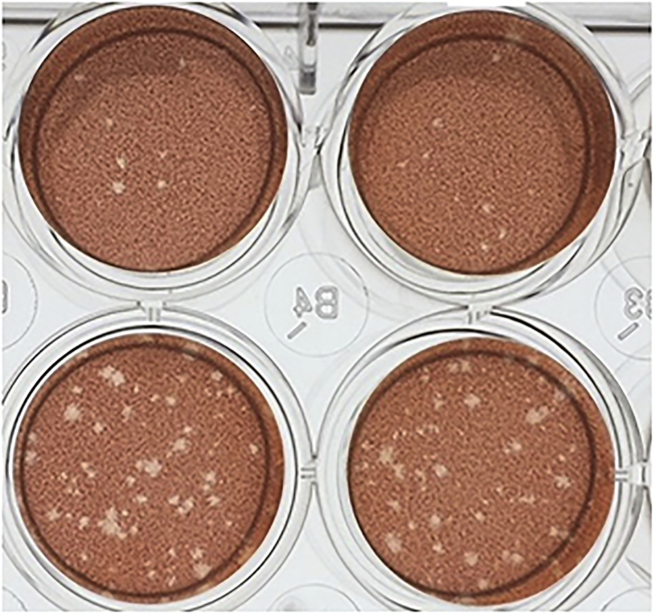
Wells of Vero cells showing plaques of dead cells, i.e. not stained by the red colour, caused by SINV. The two wells on the top row are duplicates of a sample reducing the number of plaques ≥80%. while the two wells on the bottom row are duplicates of a sample with <50% plaque reduction. (For interpretation of the references to colour in this figure legend, the reader is referred to the web version of this article.)

On day two, 500 μL of 5% Neutral red solution (Sigma-Aldrich, MO, USA) diluted in PBS was added to each of the wells and incubated over night. On day three, the Neutral red was removed and all plaques were counted (Fig. 2). The mean number of plaques were calculated from each sample pair as well as from the six wells containing the virus control dilution. The plaque neutralisation percent was calculated as: 1- (mean of sample wells/mean of virus control dilution wells).

Samples showing a high degree of neutralisation were also sequentially two-fold titrated before being mixed with virus and tested as above, to allow determination of the end-point serum dilution required to neutralise the virus.

## 3 Results

SINV-PRNTs were performed on 171 serum samples collected from horses in 20 regions in Sweden between spring and autumn, 2019 (Fig. 1). The measured plaque reduction ranged between 0 and 99% (Table 1), with six horses having serum that reduced SINV plaques 80% or more (≥PRNT_80_). End-point titration of these samples showed that the maximum serum dilution for ≥PRNT_80_ was 40×.

**Table 1:** Reduction of SINV plaques by horse serum samples collected in Sweden 2019, as compared to the virus dilution control.

Plaque reduction percent	No. of horses	Percent of total horses tested
0–9	7	4%
10–19	3	2%
20–29	13	8%
30–39	25	15%
40–49	39	23%
50–59	45	26%
60–69	29	17%
70–79	4	2%
80–89	4	2%
90–99	2	1%
Total	171	

Four of the six horses with ≥PRNT_80_ were collected in early summer, while two were collected in mid/late-summer. Three of the horses were sampled in regions previously known to be highly-endemic for SINV (Värmland, Dalarna, Västernorrland), while three were sampled in other regions (Table 2).

**Table 2:** Sampling date and location of horses that reduced SINV plaques ≥80%. All horses were sampled in 2019.

Sampling month	No. of horses tested	< 80% reduction	80–89% reduction	90–99% reduction	Date and location of ≥PRNT_80_ horses
April	14	13	–	1	18 April, Värmland
May	18	17	1	–	15 May, Västernorrland
June	34	33	1	–	10 June, Uppsala
July	54	52	2	–	4 July, Stockholm 29 July, Kronoberg
August	38	37	–	1	12 August, Dalarna
September	12	12	–	–	NA
October	1	1	–	–	NA

NA: Not Applicable.

## 4 Discussion

In this study we detected high reduction of SINV-I (≥PRNT_80_) in serum from six horses in Sweden, indicating a preceding SINV infection. Previously, SINV-I infection in horses has been reported from South Africa, where SINV RNA was detected in the brains of horses showing neurological symptoms [11]. South African SINV-I strains are closely related to SINV-I strains that are endemic in some parts of Sweden, indicated by high infection rates in mosquitoes (3.6% of the *Culex* population carrying the virus in some years) and high antibody prevalence in passerine birds (reaching 78% in some species) [14,15]. Clearly SINV-I is commonly transmitted between bird-biting mosquitoes and birds in these regions, but tangential transmission to alternative hosts, such as humans and horses, is less described and understood.

Another report of SINV neutralising antibodies in one horse and one donkey, originates from Australia where two other SINV genotypes circulate (SINV-II and SINV-VI) [5]. The authors used Whataroa virus for their assays, which is the same as SINV genotype V, detected only in New Zealand, but they do not exclude it might have been a reaction to the SINV genotypes circulating in Australia. Niklasson et al. [16] showed that such cross-reactions from antisera between different SINV genotypes can occur. Thus, it is possible that several SINV genotypes may cause infection in horses.

Reported cases of human SINV infection in Sweden are very few, usually ranging between none and ten per year [18]. In some years, however, the number of human cases increase; e.g. 60 cases were diagnosed in the small town of Lövånger in Västerbotten in 2012 [19]. Significant yearly variations in virus prevalence are also evident from virus detection in the mosquito vector population and from the antibody prevalence in the bird population [14,18]. Thus, in addition to sporadic cases, local outbreaks of SINV disease can occur, similar to many other arboviruses [20,21]. Factors affecting the risk of SINV outbreaks in humans or horses are so far unknown.

The sampling location of some of the ≥PRNT_80_ horses (e.g. Kronoberg), are south of the previously established main endemic region for SINV in Sweden [9]. Human disease cases and antibodies against SINV have been detected also in southern Sweden, although at a lesser extent, thus transmission in these regions may be less intense but is not surprising. Further, any travel history of the horses has not been documented in this study and therefore sampling location is not necessary equal to where infection took place.

As this is, to our knowledge, the first study specifically conducted on SINV serology in horses, we present the whole scale of plaque reduction in Table 1. Without a deeper investigation into clinical symptoms a well-defined threshold of reduction that defines specific SINV neutralisation cannot yet be established. This clinical connection would need to include both the time of sampling and the clinical history of the horses, as the duration of the antibody response is unknown. In humans, Vene et al. [22]. show that patients developed IgM during the first two weeks after onset of symptoms, and IgG antibodies two to four weeks after onset of symptoms. In human patients, it has been shown that antibodies of both IgM and IgG class can neutralise SINV [17]. In human patients both IgM and IgG, as well as neutralising antibodies have been detected up to three to four years post infection [17,22]. In horses, studies on antibody kinetics of other alphaviruses show that IgM antibodies against RRV can sometimes be detected whilst the horses display symptoms, and neutralising antibodies against GV can be detected six days post infection [7,23,24]. IgG antibodies against RRV were still high five weeks after the onset of symptoms and neutralising antibodies against GV remained at high levels six months after infection [7,23,24]. Thus, the neutralising antibodies detected in the present study could have been produced either from relatively new infections or from infections taking place in previous seasons. As for most arboviruses the highest transmission intensity of SINV occurs after late July. However, hatchling birds have been found with SINV antibodies on June 21st, indicating occurrence of SINV transmission also earlier in the season, at least among birds [14].

Plaque neutralisation tests are the gold standard in serology, and are often used to confirm positive results from screening assays such as ELISAs, immunofluorescence and immunoblotting assays [25]. ELISA systems are often prone to cross-reactions between closely related viruses, but are much less labour intensive than neutralisation tests. Unspecific reactions are common in all laboratory testing systems, and therefore neutralisation tests often have a cut off value of 50%, 80% or 90% reduction in plaque count to define virus specific neutralisation [[25], [26], [27]]. Previous studies on SINV neutralising antibodies in humans and birds have used an 80% reduction as cut-off [14,16,17,28]. In our study, plaque reduction varied between 0 and 99%, with half of the sera reducing plaques 40–60% (Table 1). No other alphavirus is known to infect horses in Sweden, thus sera reducing plaques more than 80% have very likely been infected with SINV, although no conclusions on development of disease can be drawn from this study. The other arboviruses known to be present in Sweden are flaviviruses and orthobunyaviruses, which would not cross-react in a SINV PRNT [28,29]. Tick-borne encephalitis virus (TBE) is known to cause infection in horses, but is not routinely tested for in Swedish horses since the connection to disease is poorly understood [30]. Other flaviviruses that circulate in central and southern Europe and known to cause infection in horses are WNV and Usutu virus (USUV), but reports of these viruses from Sweden are very few [31,32] and it is not established whether they are circulating in the country [13].

As with most arboviruses, horses and humans are believed to be dead-end hosts of the virus, meaning that they cannot produce high enough viremias in their blood to infect new mosquitoes. However, very few studies have actually investigated the viremias in humans and horses and compared them with mosquito infection requirements. In this study we show that horses in Sweden have neutralising antibodies against SINV-I, which suggests for an increased awareness of the potential of SINV caused disease in horses. In addition, if horses are commonly infected with SINV without developing disease they may be utilized as sentinels for epidemic transmission outside the usual bird-mosquito-bird transmission cycle.

## Funding

This work was supported by The 10.13039/501100003748Swedish Society for Medical research, the 10.13039/501100002805Carl Trygger foundation, and the 10.13039/501100000780European Union‘s Horizon 2020 research and innovation programme under grant No. 874735 (VEO).

## Author's contribution

All authors have read and commented on the manuscript.

## Competing interest

The authors declare that they have no competing interest.
